# Hip and Knee Osteoarthritis in Patients with Chronic Myeloproliferative Neoplasms: A Cross-Sectional Study

**DOI:** 10.3390/life13061388

**Published:** 2023-06-14

**Authors:** Hrvoje Holik, Ivan Krečak, Marko Lucijanić, Ivan Samardžić, Danijel Pilipac, Ivana Vučinić Ljubičić, Božena Coha, Alma Kitter Pipić, Blaženka Miškić, Silva Zupančić-Šalek

**Affiliations:** 1 Department of Internal Medicine, Dr. Josip Benčević General Hospital, 35000 Slavonski Brod, Croatia; 2 Faculty of Medicine, Josip Juraj Strossmayer University of Osijek, 31000 Osijek, Croatia; 3 Department of Internal Medicine, General Hospital of Šibenik-Knin County, 22000 Šibenik, Croatia; 4 Faculty of Medicine, University of Rijeka, 51000 Rijeka, Croatia; 5 University Hospital Dubrava, 10000 Zagreb, Croatia; 6 Faculty of Medicine, University of Zagreb, 10000 Zagreb, Croatia; 7 Department of Orthopaedic Surgery, Dr. Josip Benčević General Hospital, 35000 Slavonski Brod, Croatia; 8 Department of Orthopaedic Surgery, General Hospital of Šibenik-Knin County, 22000 Šibenik, Croatia; 9 Department of Laboratory Diagnostics, General Hospital ‘Dr Josip Benčević’, 35000 Slavonski Brod, Croatia; 10 Faculty of Dental Medicine and Health Osijek, Josip Juraj Strossmayer University of Osijek, 31000 Osijek, Croatia; 11 Department of Hematology and Coagulation, University Hospital Holy Spirit, 10000 Zagreb, Croatia

**Keywords:** osteoarthritis, bone pain, myeloproliferative neoplasms, JAK2-V617F, cytoreductive treatment

## Abstract

Background: Osteoarthritis (OA) is a progressive degenerative disease with an inflammatory background. Chronic myeloproliferative neoplasms (MPN) are clonal hematopoietic disorders characterized by chronic inflammation and a tendency for connective tissue remodeling. Aim: This study aimed to investigate the prevalence and associated risk factors of symptomatic OA (sOA) in MPN patients. Patients and methods: A total of 100 consecutive MPN (39 essential-thrombocythemia, 34 polycythemia-vera, 27 myelofibrosis) patients treated in two community hematologic centers were cross-sectionally evaluated. Patients were required to have both symptoms attributable to hip and/or knee OA and radiographic confirmation to be considered as having sOA. Results: The prevalence of hip and/or knee sOA was significantly higher among MPN patients than the previously reported prevalence in the general population of similar age (61% vs. 22%, *p* < 0.001). Hip sOA was present in 50%, knee sOA in 51% and sOA of both localizations in 41% of patients. A high proportion of MPN patients had radiographic signs of hip OA (94%) and knee OA (98%) in the presence of attributable symptoms. Among the other factors, sOA was univariately associated with the presence of *JAK2* mutation, myelofibrosis phenotype, older age, higher body weight, and higher MPN-SAF score (*p* < 0.050 for all analyses). In the multivariate analysis, older age (odds ratio = 1.19, 95% confidence interval-CI 1.06–1.33) and higher body weight (OR = 1.15, 95% CI 1.06–1.25) were recognized as independent risk factors for sOA. On the other hand, cytoreductive treatment was a protective factor for sOA (OR = 0.07, 95% CI 0.006–0.86). Conclusions: The prevalence of sOA in MPN patients was higher than that in the general population and seems to correlate with older age, increased myeloproliferation and a higher inflammatory state. Whether cytoreductive treatment may postpone OA development in MPN patients warrants additional confirmation.

## 1. Introduction

Osteoarthritis (OA), the most common form of arthritis, is a progressive degenerative disease that affects all components of the joint: cartilage, subchondral bone, ligaments, joint capsule, and synovial membrane [1,2,3]. The pathogenesis of OA is complex and includes multiple risk factors such as aging, obesity, inflammation, mechanical, and different metabolic aberrations [4,5]. OA can affect any joint, but the hips, knees, and hands are the most commonly affected ones [5]. The prevalence of radiographic knee and hip OA are up to 28% [6,7,8,9,10]. On the other hand, symptomatic OA (sOA) and radiologically confirmed OA have a much lower incidence (up to 17% for knee OA and 9% for hip OA) because radiographic OA can also be asymptomatic [11].

sOA may significantly affect the quality of life and cause increased socioeconomic costs worldwide [12]. The main treatment goals are to reduce pain, to improve joint function, and to slow down OA progression [1,12,13,14,15,16]. Currently, there are no specific treatments that can repair the joint damage caused by OA. Body weight reduction, regular exercise, and occasional analgesic use are the most commonly prescribed treatments. In most severe cases, however, total hip or knee arthroplasty is needed. 

Patients with sOA have higher circulating values of different proinflammatory cytokines when compared to the general population [17,18,19]; the most important ones are interleukin (IL)-1, tumor necrosis factor (TNF)-α, IL-6, IL-15, IL-17, and IL-18 [20]. In fact, there is growing evidence suggesting that low-grade inflammation is extremely important for the development and progression of OA [17,18,19,20,21]. Moreover, it seems that the presence of metabolic syndrome with a high inflammatory milieu may be more important for the pathogenesis of OA than obesity alone [17,18].

Philadelphia chromosome-negative myeloproliferative neoplasms (MPNs), polycythemia vera (PV), essential thrombocythemia (ET), and primary myelofibrosis (PMF) are clonal hematopoietic stem cell disorders characterized by excessive myeloproliferation of at least one myeloid lineage (granulocytic, erythroid, or megakaryocytic), variable degrees of bone marrow fibrosis, chronic inflammation, and high cardiovascular risk [22,23].

MPN are disorders of the elderly (the median age at the time of disease diagnosis is 60 years) and have an incidence of 1 to 2/100,000 per year in Europe [24,25]. Even though life expectancy of all three MPN is worse than that of the general population [26,27,28], many ET and PV patients may experience a normal lifespan [29]. Therefore, the prevalence of MPNs is much higher and is approximately 80–100/100,000 [26,27]. On the other hand, MF has the most aggressive behavior, with a median overall survival of approximately 5 years [30]. The most common disease complications in all three MPN are thrombohemorrhagic events and disease transformation to secondary (post-ET and post-PV) MF, myelodysplastic syndrome, and acute leukemia [31].

MPN are clonally driven by mutually exclusive mutations in the Janus kinase 2 (*JAK2*), calreticulin (*CALR*), or myeloproliferative leukemia virus (*MPL*) genes [32]. These mutations cause constitutive activation of the signal transduction pathway (*JAK-STAT*), leading to a chronic inflammatory state that promotes clonal expansion and disease progression and causes frequent debilitating symptoms associated with the disease, such as bone pain, weight loss, night sweats, itching, fever, lack of concentration, or loss of libido [23,33]. Several studies have demonstrated high serum concentrations of different proinflammatory cytokines in MPN patients, including IL-2R, IL-4, IL-8, IL12, IL-15, granulocyte macrophage-colony stimulating factor, interferon-γ, monocyte chemotactic protein-1, platelet derived growth factor-β, and YKL-40 [34,35,36,37], some of which may be responsible for the osteoarticular damage [1,2,3]. It should also be pointed out that the inflammatory response associated with the *JAK2* mutation is biologically different and is much stronger when compared to the *CALR*-mutated one [38]. Finally, this cytokine-driven tissue remodeling is not only responsible for the development of bone marrow fibrosis [39] but may also be responsible for the increased bone morbidity in MPN [40].

Considering that MPNs are disorders typically diagnosed in the elderly and are characterized by excessive myeloproliferation, high cardiovascular risk, chronic inflammation, and tissue remodeling, this study aimed to investigate the prevalence of hip and knee sOA in MPNs and its associated risk factors.

## 2. Patients and Methods

### 2.1. Study Design

This was a cross-sectional observational study conducted in the period between October 2021 and March 2023. Relevant demographics (age at study entry, sex, body height, body weight, and body mass index-BMI), clinical (MPN disease characteristics, comorbidities, Eastern Cooperative Oncology Group-ECOG performance status, and treatments) and laboratory data (complete blood counts and biochemical, inflammatory and bone turnover biomarkers), as well as the MPN Symptom Assessment Form (MPN SAF), were taken at the time of study entry. All MPN patients were evaluated by an orthopedic surgeon and those with suspected hip and/or knee sOA (asymmetry, pains, swelling, crackling, or reduced flexibility) underwent X-ray imaging of the symptomatic joint(s). Patients with unilateral joint pain underwent bilateral joint X-ray imaging, and the presence of either uni- or bilateral sOA was considered as a positive finding. 

### 2.2. Setting

The study was conducted in two community hospitals in Croatia (Dr. Josip Benčević General Hospital, Slavonski Brod and General Hospital of Šibenik-Knin County, Šibenik, Croatia).

### 2.3. Participants

Consecutive patients with ET, PV as well as MF ET, PV and MF diagnosed according to 2016 World Health Organization criteria [23], and patients with post-ET and post-PV MF diagnosed according to International Working Group for Myelofibrosis Research and Treatment criteria [41] were offered to participate in the study. Excluded from participation were MPN patients younger than <18 years of age, pregnant women, those with prior hip or knee trauma or joint infection, patients with other cancers, and subjects with occupational risk factors for sOA (i.e., workers in construction, firefighting, agriculture, and forestry). 

### 2.4. Definitions of Measured Variables

Radiographic OA was defined as grade ≥1 according to the Kellgren–Lawrence (KL) classification [42] in order to capture the earliest joint changes. More specifically, this classification classifies OA into 5 stages: stage 0 represents normal joint, stage 1 indicates suspected OA (doubtful narrowing of joint space and possible osteophytic lipping), stage 2 mild OA (definite osteophytes and possible narrowing of joint space), stage 3 moderate OA (moderate multiple osteophytes, definite narrowing of joint space, some sclerosis, and possible deformity of bone ends), and stage 4 severe OA (large osteophytes, marked narrowing of joint space, severe sclerosis, and definite deformity of bone ends). We did not use the common American College of Rheumatology (ACR) criteria for hip [43] and knee OA [44] because the KL criteria may detect earlier osteoarticular changes than the ACR [45]. In addition, the majority of MPN patients are older than 50 years of age [24,25], and they often have a low erythrocyte sedimentation rate due to increased myeloproliferation [46], which could have made ACR less reliable in this particular patient population. 

The MPN SAF is a prospectively validated questionnaire used to measure symptom burden in MPN patients; this particular tool is used to assess patient symptoms over time and to guide treatments and has 10 items focusing on fatigue, concentration, early satiety, inactivity, night sweats, itching, bone pain, abdominal discomfort, weight loss, and fever. Each score ranges from 0 (absent/as good as it can be) to 10 (worst imaginable/as bad as it can be), and the maximum possible score is 100 [47]. 

Estimation of glomerular filtration rate (eGFR) was performed using the Modification of Diet in Renal Disease (MDRD) formula [48]; chronic kidney disease (CKD) was defined as eGFR < 60 mL/min/1.73 m^2^ ≥ 3 months [49].

Complete blood counts were analyzed with Siemens Advia 120 and 2120i (Siemens Medical Solutions Diagnostics Pte Ltd., Swords, Ireland) and Sysmex XN-1000 analyzers (Sysmex Europe GMBH, Norderstedt, Germany). Osteocalcin and β-crosslaps were assessed on Cobas 6000 analyzer (Roche Diagnostics, Mannheim, Germany). Other biochemical variables were assessed with Abbott Alinity (Abbott Laboratories, Chicago, IL, USA) analyzer.

### 2.5. Sample Size

With a type one error set at 0.05, 80% power, and the expected increase in the prevalence of 50% compared to the general population, a total of 86 patients were required to detect statistical significance.

### 2.6. Statistics

Statistical analyses were performed with MedCalc Statistical Software^®^ (version 20.216, Ostend, Belgium). The distribution of our data was checked using the Shapiro–Wilk test. A test for one proportion was used to assess the differences in the observed and expected sOA prevalence. Categorical variables were compared using the chi-square test, and quantitative variables were compared with the Mann–Whitney U test or the Student’s t-test, as appropriate. Multivariate logistic regression analysis was used to identify its associated risk factors. *p* values of <0.050 were considered statistically significant for all presented analyses.

## 3. Results

We included 100 MPN patients (39 ET, 34 PV, and 27 MF [17 PMF, 3 post-PV MF, and 7 post ET-MF]); the median age was 68 years (range, 35–90) and 56 (56%) were female. Patient clinical and laboratory characteristics are summarized in Table 1 and Table 2, respectively. 

**Figure 1:**
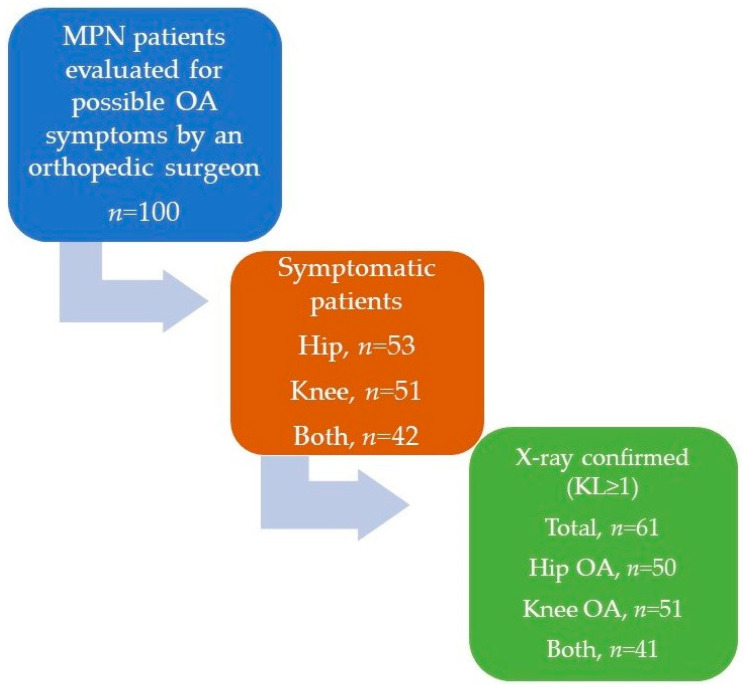
Brief study flowchart summarizing patient workup. MPN = myeloproliferative neoplasm, OA = osteoarthritis, KL = Kellgren–Lawrence classification.

A total of 53 (53%) individual MPN patients had hip-related and 52 (52%) had knee-related symptoms that could be attributable to sOA, with a high proportion of patients (42%) with both hip and knee OA-attributable symptoms presenting synchronously. Out of 53 patients with hip-related symptoms, 50 (94.3%) had radiographic signs (KL score ≥1) of hip OA (*p* < 0.001). Out of 52 patients with knee-related symptoms, 51 (98.1%) had radiographic signs of knee OA (*p* < 0.001). Out of 42 patients with both hip- and knee-related symptoms, 41 (97.6%) had radiographic signs at both localizations (*p* < 0.001). A brief study flowchart summarizing the patient workup is presented in Figure 1. 

**Figure 2:**
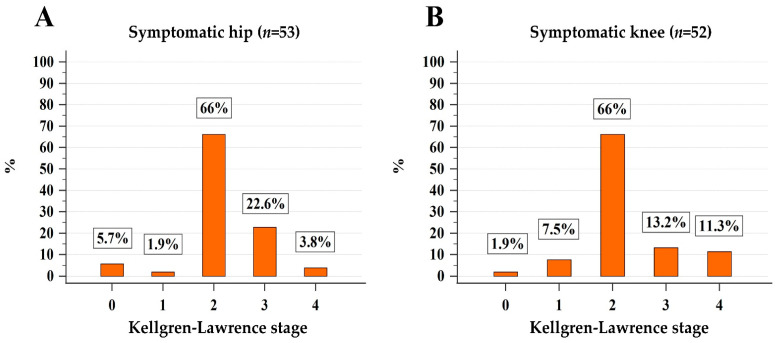
Stratification of patients with symptoms attributable to hip (**A**) and knee (**B**) osteoarthritis according to the Kellgren–Lawrence classification.

As shown in Figure 2, only 1.9% (*n* = 1) and 7.5% (*n* = 4) of sOA patients had stage 1 radiographic hip and knee OA, respectively, and the vast majority of patients had radiographic ≥2 stage hip (49/53, 92.4%) or knee OA (48/53, 90.5%). Fourteen and 13 patients had moderate/severe hip and knee OA, respectively. Finally, sOA of hip or knee was present in 61 (61%) of MPN patients which seems to be significantly higher than the expected one (22%) in the general population of similar age (median age of 75 years) [10] (*p* < 0.001). 

As shown in Table 1, MPN patients with sOA were more often older (*p* < 0.001), with MF phenotype (*p* = 0.034) and *JAK2* mutation (*p* = 0.029), more often had worse ECOG performance status (*p* = 0.006), history of thrombosis (*p* = 0.035), higher body weight (*p* = 0.001) and higher BMI (*p* < 0.001), arterial hypertension (*p* = 0.029), CKD (*p* = 0.029), and hyperlipidemia (*p* = 0.014), and they more frequently used non-steroidal antirheumatic drugs (*p* = 0.021), and opioids (*p* = 0.032), and had lower hemoglobin (*p* = 0.004) and hematocrit levels (*p* = 0.029), higher red cell distribution width (RDW; *p* = 0.027), higher serum uric acid (*p* = 0.049), lower eGFR (*p* = 0.007), lower total cholesterol (*p* = 0.044), low-density lipoprotein (*p* = 0.019), and lower high-density lipoprotein (*p* = 0.014) and had slightly lower serum calcium levels (*p* = 0.030). These patients also had higher total MPN SAF scores (*p* < 0.001) as well as higher scores for fatigue, early satiety, inactivity, concentration problems, itching, and bone pains (*p* < 0.05 for all analyses). Biomarkers of bone metabolism (osteocalcin, β-cross laps, parathormone, serum phosphate, and vitamin D) did not differ significantly between MPN patients with and without sOA (*p* > 0.05 for all analyses).

Finally, a multivariate logistic regression model with sOA of any localization as a dependent variable was created in order to identify the potential sOA risk factors in MPN patients and is presented in Table 3. Older age (odds ratio-OR 1.19, 95% confidence interval-CI 1.06–1.33) and higher body weight (OR = 1.15, 95% CI 1.06–1.25) were independently associated with the presence of sOA, whereas cytoreductive treatment (OR = 0.07, 95% CI 0.006–0.86) had protective properties; sex, MF phenotype, presence of *JAK2* mutation, arterial hypertension, hyperlipidemia, smoking, CKD, and serum uric acid lost their independent associations with sOA in the multivariate analysis.

## 4. Discussion

Our results revealed a high prevalence (61%) of symptomatic and radiologically proven OA in MPN patients. Previous studies from the general population [6,7,8] demonstrated a much lower prevalence of symptomatic and radiologically confirmed OA of 17% and 9% for knee and hip OA, respectively. In fact, even though the general population from Germany was slightly older (median age of 75 years) when compared to our study population (median age of 68 years), the prevalence of symptomatic hip and/or knee OA was significantly higher in MPN patients (61% vs. 22) [10]. Moreover, 14% and 13% of all MPN patients included in the study had symptomatic and radiographic moderate/severe hip and knee OA, respectively. It is also worth noting that almost all symptomatic MPN patients included in our study had radiographic signs of OA (94.3% and 98.1% concordance of symptoms and radiographic findings for hip and knee OA, respectively). These findings suggest that timely detection of early joint changes in MPN may help to promptly recognize early OA with the aim of timely utilization of different therapeutic strategies (i.e., weight loss, balanced physical activity, stringent cardiovascular risk factor control, orthopedic surgery consultations, etc.), which could help to slow down the progression of OA. However, further studies are needed to evaluate whether other more powerful imaging techniques (i.e., magnetic resonance-MR or computed tomography-CT) may show even earlier and possibly more specific, “MPN-related”, morphological changes of the joints [9], as well as whether MPN specific therapies may positively affect OA course and its associated complications. 

In our study, we found a similar prevalence of hip and knee sOA (50% and 51%, respectively), whereas the prevalence of knee sOA seemed to be slightly higher than that of the hip sOA in the general population [6,7,8]. It should be pointed out, however, that hip and knee OA differ not only in prevalence but also according to other important disease characteristics–pathogenesis, epigenetics, clinical presentation, and prognosis [50]. It is possible that the influence of MPN somehow contributed to the equalization of these differences, given the high but similar prevalence of hip and knee sOA in our cohort; however, additional research in this direction is needed. Interestingly, even though OA is somewhat more common in women [2], our study found no such difference, although the proportion of women was slightly higher (59% vs. 51% for MPN patients with and without sOA, respectively). Importantly, MPN patients with sOA used more non-steroidal antirheumatic drugs (NSAIDs) and opioids for symptom relief. In fact, the proportion of MPN patients with sOA who used NSAIDs in our study was quite similar to that from the German study [10], emphasizing the high OA-driven symptom burden in MPN patients.

As expected, the presence of sOA in MPN patients was associated with established risk factors for OA development in the general population, such as advanced age, increased body weight, and increased BMI. More interestingly, parameters suggestive of stronger myeloproliferation and more aggressive disease, such as MF phenotype, *JAK2* mutation, higher total MPN SAF score, lower performance status, thrombosis history, lower hemoglobin and hematocrit values, and higher RDW, were more frequent in MPN patients with sOA. It should be pointed out that, when compared to ET and PV, MF patients have the shortest survival and the highest rate of transformation into acute leukemia; these patients also usually have a higher total MPN SAF score, lower hemoglobin, hematocrit, and higher RDW values [51]. Additionally, the presence of prior thrombosis in ET and PV also classifies patients as those at high risk [32], and more thrombotic events were recorded in MPN patients with sOA than in those without. Moreover, the *JAK2* mutation is associated with a higher frequency of thrombosis, lower survival, and a more pronounced inflammatory response in MPN [38,52,53,54,55,56]. *JAK2*-mutated patients univariately had a higher frequency of sOA when compared to their *CALR-* and *MPL*-mutated counterparts. However, the association of the *JAK2* mutation with the presence of sOA was lost in the multivariate analysis, probably due to the small number of non-*JAK2* mutated patients in the study. Therefore, additional research on a larger number of subjects is needed to elucidate whether the presence of *JAK2* mutation *per se* may indeed be associated with an increased risk of developing sOA in MPN and in the general population as well (i.e., as a biomarker of clonal hematopoiesis of indeterminate potential–CHIP). 

The RDW was also higher in MPN patients with sOA, and this particular biomarker has been shown to be a sign of a more aggressive disease [57,58]. On the other hand, the ratio of neutrophils to lymphocytes (NLR) and the ratio of platelets to lymphocytes (PLR) were recently shown to be reliable biomarkers of increased myeloproliferation and systemic inflammation in MPN and to be associated with worse outcomes in MPN if increased [59,60,61]. In contrast to RDW, however, NLR and PLR were not associated with the presence of sOA in MPN, possibly due to the fact that higher RDW may better reflect the presence of comorbidities than NLR and PLR in MPN patients [57,58,59,60,61]. Similarly, the MPN SAF questionnaire excellently assesses the disease burden and the presence of inflammation-linked symptoms in MPN [43,62,63]. In the present study, MPN patients with sOA had significantly higher total MPN SAF scores and several disease-specific symptoms (fatigue, early satiety, inactivity, concentration problems, itching, and bone pain) were more pronounced in sOA patients, indicating a higher symptomatic disease burden present in this subset of patients.

Our results also demonstrate that cytoreductive treatment may have a protective role for sOA development in MPN patients. This important observation suggests that adequate control of the MPN clone with cytoreductive treatment may also have favorable effects on the osteoarticular system. It should also be pointed out that the majority of MPN patients included in the study were treated with HU; a similar protective effect of HU on the osteoarticular system was also found in patients with sickle cell anemia [64]. Considering that early therapeutic intervention (especially with interferons) in MPN has recently been put into limelight [65,66], future prospective studies are needed to investigate whether other cytoreductive treatments may also possess similar activity, and whether their early initiation in MPN may postpone OA development and progression.

The presence of arterial hypertension and hyperlipidemia were univariately associated with an increased prevalence of sOA in MPN patients, which is in line with the current knowledge in the field [67,68], but these associations were lost in the multivariate analysis. More interestingly, the presence of sOA correlated with lower serum lipids, which could be explained with the influence of statin therapy, but also due to the high membrane uptake of cholesterol caused by an increased cell turnover in the bone marrow, potentially suggesting a more aggressive disease biology in MPN patients with OA [68,69]. Considering that statins may exert beneficial effects on both the osteoarticular system [70] and the MPN clone [71,72], additional studies are needed to unravel whether these compounds may also potentially have beneficial effects on OA development and progression in MPN patients. 

Interestingly, smokers more often had sOA; however, this observation was not confirmed in multivariate analysis. The controversy regarding the effect of smoking on sOA development and progression indeed exists [73,74,75], and studies on larger numbers of MPN patients are needed to clarify whether smoking has an effect on OA development in MPN patients. This may be even more important in light of the recent findings indicating that smoking may be responsible for MPN development, lower treatment responses, and impaired survival [76,77].

Even though there was no significant difference with respect to gout history, MPN patients with sOA had higher serum uric acid levels. Similarly, the prevalence of CKD was also higher and eGFR was lower in sOA patients when compared to those without sOA, but these associations were also lost in the multivariate analysis. Interestingly, both serum uric acid and CKD have recently been associated with parameters suggestive of stronger myeloproliferation and inflammation in MPN patients. More worrisome, MPN patients with lower eGFR and higher serum uric acid levels also had a higher frequency of thrombosis and death [78,79]. Considering the controversial role of serum uric acid in sOA development [80,81,82] and its close relationship with CKD [83], additional studies on MPN patients are needed to unravel whether serum uric acid and/or CKD may represent a potentially treatable target in MPNs (i.e., with uricosurics or cytoreductive treatments) [84] and whether this therapeutic approach may be associated with a lower frequency of sOA. 

Finally, even though population-based studies have demonstrated reduced bone density and an increased tendency to osteoporotic fractures in MPN patients [40], there were no significant differences in the bone metabolism biomarkers in patients with MPNs with or without sOA in our study. Osteocalcin, β-cross laps, parathormone, serum phosphate, and vitamin D were equal in both studied populations, suggesting that bone metabolism turnover in MPN patients may not be the most important pathophysiological mechanism for sOA development. However, it should be noted that there are also data showing no evidence of secondary osteoporosis in patients with MPNs [85]. 

The limitations of this study are the relatively small number of patients included, the heterogeneous population with respect to particular disease subtypes, the lack of assessment of inflammatory biomarkers that could be associated with sOA development in MPN patients, and the absence of other imaging modalities (i.e., bone densitometry, scintigraphy, or MR and/or CT scans of the hip and knees) for the investigation of sOA. Additionally, even though we intentionally classified radiographic OA as Kellgren–Lawrence stage ≥1 in order to capture the earliest joint changes in this specific patient population, the majority of symptomatic patients (>90%) had stage ≥2, confirming the unusually high prevalence of sOA in MPNs. Finally, considering the low number of MPN patients with stages one, three, and four OA, we did not have enough statistical power to analyze clinical and laboratory differences among different OA stages. 

## 5. Conclusions

The results of this study highlight the significant osteoarticular morbidity in MPN patients and emphasize the importance of early sOA recognition in this particular patient population. We have identified advanced age and higher body weight to be independent risk factors for sOA development in MPN patients, whereas cytoreductive treatment (predominantly with HU) had a protective effect. Therefore, timely recognition of sOA in MPN patients is needed in order to adequately control the underlying risk factors (especially higher body weight), which may help to delay sOA progression and the potential sOA-related disabilities as well as to mitigate associated socioeconomic and healthcare costs. Nevertheless, considering the limited number of patients included and the cross-sectional design of the study, our findings should only be considered as preliminary. In addition, the putative protective effect of cytoreductive treatment on sOA development in MPN patients warrants additional confirmation.

## Figures and Tables

**Table 1:** Patient characteristics. The chi-square test, the Mann–Whitney U test, and the t-test were used.

Variable	Overall (n = 100)	sOA (n = 61)	Non sOA (n = 39)	Difference between sOA and Non sOA Patients
Age, years (median, range)	68 (35–90)	70 (41–90)	61 (35–79)	***p* < 0.001**
Sex, female	56	36 (59%)	20 (51.3%)	*p* = 0.449
Disease duration, years (median, range)	2 (0–20)	2 (0–20)	2 (0–15)	*p* = 0.827
Newly diagnosed	25	16 (26.2%)	9 (23.1%)	*p* = 0.723
Overall				***p* = 0.397**
ET	39	21 (53.8%)	18 (46.2%)	*p* = 0.631
PV	34	21 (61.8%)	13 (38.2%)	*p* = 0.170
MF	27	19 (70.4%)	8 (29.6%)	*p* = 0.034
Driver mutation				
JAK2-V617F	78	52 (85.2%)	26 (66.7%)	***p* = 0.029**
CALR	13	5 (8.2%)	8 (20.5)	***p* = 0.078**
Negative	9	3 (4.9%)	6 (15.4)	***p* = 0.075**
Total MPN-SAF score (median, range)	17 (0–50)	20 (0–50)	11 (0–43)	***p* = <0.001**
ECOG (2–4)	22	19 (31.1%)	3 (7.7%)	***p* = 0.006**
Prior thrombosis	21	17 (27.9%)	4 (10.3%)	***p* = 0.035**
Body weight (kg)	77.4 (±14.08)	81.4 (±13.4)	70.6 (±12.5)	***p* = 0.001**
Body height (m)	1.69 (±8.8)	169.4 (±9.1)	168.9 (±8.4)	*p* = 0.818
BMI, kg/m^2^	26.9 (±4.2)	28.3 (±3.9)	24.6 (±3.6)	***p* = <0.001**
Arterial hypertension	62	43 (70.5%)	19 (48.7%)	***p* = 0.029**
Diabetes mellitus	11	9 (14.8%)	2 (5.1%)	*p* = 0.135
Chronic kidney disease (n = 99)	25	20 (32.8%)	5 (13.2%)	***p* = 0.029**
Hyperlipidemia	38	29 (47.5%)	9 (23.1%)	***p* = 0.014**
Smoking	22	9 (14.8%)	13 (33.3%)	***p* = 0.029**
Autoimmune disorders	2	2 (3.3%)	0	*p* = 0.255
Gout history	6	5 (8.2%)	1 (2.6%)	*p* = 0.249
Cytoreduction	77	47 (77%)	30 (76.9%)	*p* = 0.988
Hydroxycarbamide (n = 72)				
Interferons (n = 4)				
Ruxolitinib (n = 1)				
Low-dose aspirin	78	46 (75.4%)	32 (82.1%)	*p* = 0.436
Oral anticoagulants	12	10 (16.4%)	2 (5.1%)	*p* = 0.092
NSAIR	37	28 (45.9%)	9 (23.1%)	***p* = 0.021**
Opioids	18	15 (24.6%)	3 (7.7%)	***p* = 0.032**
Corticosteroids	4	4 (6.6%)	0	*p* = 0.108

Statistically significant *p*-values are bolded and set to < 0.050. sOA = symptomatic osteoarthritis, ET = essential thrombocythemia, PV = polycythemia vera, MF = myelofibrosis, MPN = SAF = Myeloproliferative Neoplasms Symptoms Assessment Form, ECOG = Eastern Cooperative Oncology Group, NSAIR = Non-steroidal antirheumatics, ACE-I = angiotensin-converting enzyme inhibitors, and KL = Kellgren–Lawrence.

**Table 2:** Patient laboratory values. The chi-square test, the Mann–Whitney U test, and the t-test were used.

Variable	Overall (n = 100)	sOA (n = 61)	Non sOA (n = 39)	Difference between sOA and Non sOA Patients
Total leukocytes (×10^9^/L)	7.6 (2.9–24)	7.6 (2.9–24)	8.1 (3.8–14.5)	*p* = 0.615
Granulocytes (×10^9^/L)	5.2 (1.5–17.4)	5.1 (1.5–17.4)	5.3 (1.8–13.2)	*p* = 0.694
Lymphocytes (×10^9^/L)	1.6 (0.7–4.4)	1.45 (0.7–4.4)	1.6 (0.7–4)	*p* = 0.598
Hemoglobin (g/L)	139 (79–192)	136 (79–192)	142 (86–162)	***p* = 0.004**
Hematocrit (%)	43 (25–61.8)	42.5 (25–61.8)	44 (27–59)	***p* = 0.029**
Platelets (×10^9^/L)	407 (56–1293)	401.5 (56–1293)	469 (133–794)	*p* = 0.229
NLR	3.4 (1.1–14.7)	3.4 (1.1–13)	3.5 (1.1–14.7)	*p* = 0.862
PLR	270.5 (40–994.6)	262.2 (40–994.6)	284 (68.75–794)	*p* = 0.571
RDW (%)	15.4 (11.5–27.6)	16.1 (12.2–27.6)	14.5 (11.5–24.5)	***p* = 0.027**
LDH (IU/L)	226 (123–913)	231 (123–913)	212 (173–823)	*p* = 0.171
C-reactive protein (mg/L)	1.7 (0–28.5)	1.4 (0–28.5)	1.9 (0.3–6.2)	*p* = 0.886
Serum uric acid (μmol/L)	308 (146–680)	321 (177–680)	252 (89–517)	***p* = 0.001**
eGFR (mL/min/1.73 m^2^)	74.8 (26.1–1508)	72 (26.1–124.8)	81 (41.5–1508)	***p* = 0.007**
Albumin (g/L)	43 (23.35–74.73)	43 (23.35–74.73)	43.5 (38–49)	*p* = 0.260
Fibrinogen (g/L)	3.3 (2.1–8.9)	3.2 (2.1–8.9)	3.3 (2.4–5.6)	*p* = 0.843
Ferritin (μg/L)	51.5 (1–1117)	62 (1–814)	40 (5.5–1117)	*p* = 0.355
Total cholesterol (mmol/L)	4.73 (±1.27)	4.5 (±1.3)	5.1 (±1.1)	***p* = 0.044**
LDL (mmol/L)	2.86 (±0.97)	2.69 (±0.99)	3.20 (±0.86)	***p* = 0.019**
HDL (mmol/L)	1.27 (±0.32)	1.21 (±0.2)	1.39 (±0.3)	***p* = 0.013**
TGL (mmol/L)	1.34 (±0.56)	1.37 (±0.28)	1.29 (±0.35)	*p* = 0.489
PTH (pmol/L)	40.5 (0.77–130)	41 (0.77–126)	37.3 (3.61–130)	*p* = 0.609
Vitamin D (nmol/L)	45.9 (2.9–177)	43 (2.9–177)	49.2 (8–123)	*p* = 0.159
Serum calcium (mmol/L)	2.4 (1.1–2.9)	2.4 (1.1–2.6) ↓	2.4 (1–5–2.9)	***p* = 0.030**
Serum phosphate (mmol/L)	1 (0.65–1.5)	1 (0.65–1.5)	1 (0.7–1.4)	*p* = 0.953
Osteocalcin (g/L)	18.6 (4.5–77)	19 (7.3–77)	18.3 (4.5–58)	*p* = 0.556
Β-crosslaps (g/L)	321 (0.16–1444)	309 (0.49–1221)	335 (0.16–1444)	*p* = 0.920

Statistically significant *p*-values are bolded and set to < 0.050. sOA = symptomatic osteoarthritis, NLR = neutrophil to lymphocyte ratio, PLR = platelet to lymphocyte ratio, RDW = red blood cell distribution width, LDH = serum lactate dehydrogenase, eGFR = estimated glomerular filtration rate, LDL = low-density lipoprotein, HDL = high-density lipoproten, TGL = triglycerides, PTH = parathormone, and ↓ indicating overall lower score.

**Table 3:** Clinical and laboratory variables associated with the presence of symptomatic osteoarthritis. Multivariate logistic regression was used. Statistically significant results are bolded. MF = myelofibrosis and CKD = chronic kidney disease.

Variable	95% Confidence Interval	Odds Ratio
**Age**	**1.06–1.33**	**1.19**
Sex	0.64–15.32	3.13
**Body weight**	**1.06–1.25**	**1.15**
MF phenotype	0.36–12.50	2.14
JAK2-V617F	0.07–4.09	0.55
**Cytoreduction**	**0.006–0.86**	**0.07**
Arterial hypertension	0.03 to 1.80	0.26
Hyperlipidemia	0.64–17.52	3.35
Smoking	0.06–1.65	0.31
CKD	0.04–1.96	0.28
Serum uric acid	0.99–1.01	0.99

## Data Availability

Data are available from the corresponding author upon reasonable request.
